# Inclusion of *Camelina sativa* Seeds in Ewes’ Diet Modifies Rumen Microbiota

**DOI:** 10.3390/ani13030377

**Published:** 2023-01-22

**Authors:** Christos Christodoulou, Alexandros Mavrommatis, Dimitris Loukovitis, George Symeon, Vassilios Dotas, Basiliki Kotsampasi, Eleni Tsiplakou

**Affiliations:** 1 Laboratory of Nutritional Physiology and Feeding, Department of Animal Science, School of Animal Biosciences, Agricultural University of Athens, Iera Odos 75, 11855 Athens, Greece; 2 Department of Animal Production, Fisheries and Aquaculture, School of Agricultural Sciences, University of Patras, 30200 Messolonghi, Greece; 3 Research Institute of Animal Science, ELGO ‘DIMITRA’, Paralimni Giannitsa, 58100 Pella, Greece; 4 Department of Animal Production, Faculty of Agriculture, Aristotle University of Thessaloniki, 54124 Thessaloniki, Greece

**Keywords:** ewes, oilseeds, rumen, microorganisms, methane

## Abstract

**Simple Summary:**

Modern livestock research has focused on the evaluation of feeding strategies that led to modify the rumen microbiome to achieve optimum productivity without compromising ruminants’ physiology and health. For this reason, the supplementation of unconventional feedstuffs is extensively studied. In our study, we investigated the effect of *Camelina sativa* seeds supplementation on ewe’s rumen microbiota. Our results suggested that supplementing *Camelina sativa* seeds, especially in the highest studied level (160 g·kg^−1^ of concentrate), resulted in significant alterations in the relative abundance of the rumen microorganisms, with those reported in methanogens being considered the most promising.

**Abstract:**

Supplementing ruminant diets with unconventional feedstuffs (*Camelina sativa* seeds; CS) rich in bioactive molecules such as polyunsaturated fatty acids, may prove a potential eco-efficient strategy to manipulate rumen microbiome towards efficiency. Forty-eight ewes were divided into four homogenous groups (*n* = 12) according to their fat-corrected milk yield (6%), body weight, and age, and were fed individually with concentrate, alfalfa hay, and wheat straw. The concentrate of the control group (CON) had no CS inclusion, whereas the treated groups were supplemented with CS at 60 (CS6), 110 (CS11), and 160 (CS16) g·kg^−1^ of concentrate, respectively. Rumen digesta was collected using an esophageal tube and then liquid and solid particles were separated using cheesecloth layers. An initial bacteriome screening using next-generation sequencing of 16S was followed by specific microbes targeting with a RT-qPCR platform, which unveiled the basic changes of the rumen microbiota under CS supplementation levels. The relative abundances of Archaea and methanogens were significantly reduced in the solid particles of CS11 and CS16. Furthermore, the relative abundance of Protozoa was significantly increased in both rumen fluid and solid particles of the CS6, whereas that of Fungi was significantly reduced in the rumen particle of the CS16. In rumen fluid, the relative abundance of *Fibrobacter succinogens* and *Ruminobacter amylophilus* were significantly increased in the CS6 and CS11, respectively. In the solid particles of the CS11, the relative abundance of *Ruminococcus flavefaciens* was significantly reduced, whereas those of *Butyrivibrio proteoclasticus* and *Ruminobacter amylophilus* were significantly increased. Additionally, the relative abundance of *Selenomonas ruminantium* was significantly increased in both CS11 and CS16. Consequently, the highest CS content in the concentrate reduced the relative abundance of methanogens without inducing radical changes in rumen microorganisms that could impair ruminal fermentation and ewes’ performance.

## 1. Introduction

Within recent years, terms such as food-feed competition, sustainable resource management, protein alternatives, novel crops, and precise livestock are the hot topic of discussion among animal scientists and industry. Undoubtedly, ruminants appear to be the livestock sector where the aforementioned terms find sustainable implementation provided that the complex biochemical procedures of rumen could be processed and manipulated. The rumen is inhabited by a vastly diverse microbiome that includes both prokaryotic and eukaryotic species which synergistically promote digestion and utilization of the forage and other plant materials that non-ruminants cannot digest or utilize [1]. 

However, as a physiological function of rumen biochemistry; methane (CH_4_) is formed through the action of archaea almost all of them being methanogens. Although their role is crucial for the rumen ecosystem since they balance the hydrogen pressure, they are also responsible for the production of one of the most important greenhouse gases [1]. Beyond their environmental repercussion, it has been estimated that methane formation is responsible for energy losses of around 2–15% within the rumen [2].

In parallel, both the global population growth and the development of human living standards drive animal product demands into an unpreceded increase. Predictions forecast that the production of major grains, meat, and milk is projected to increase by 29–81% by 2050 compared with today’s levels, due to the aforementioned demands [3]. Within these demands, nutraceuticals and functional foods with high content of bioactive compounds, such as omega-3 polyunsaturated fatty acids (PUFA) have received considerable attention from both consumers and the industry [4]. Nevertheless, it is essential to evaluate mitigation strategies and advanced precision feeding techniques, considering that methane (CH_4_) emissions are expected to rise by 30% by 2050 as a result of meat and milk increasing demands [5]. 

Summarizing the aforementioned, the livestock sector is towards exploring and adopting novel strategies aiming to orchestrate the unbalanced nature of this growth in a sustainable and eco-efficient way by manipulating the microbial habitat of the rumen. Towards this direction, many dietary strategies have been proposed with the dominant being the effect of feedstuffs rich in bioactive compounds such as PUFA. Oilseeds are a rich source of PUFA and several plant secondary metabolites. The latter have also been associated with toxic/inhibitory effects on methanogenic rumen microbes [6]. Up until now, supplementing ruminant diets with oilseeds has resulted in different outcomes regarding the modification of the rumen microorganism population. For instance, rumen bacteriome were not affected by linseed oil supplementation in goats’ diets, when the total dietary ether extract content was similar to or lower than the control group [7]. Additionally, linseed supplementation did not negatively affect *Ruminococus albus*, which is one of the most vital cellulolytic bacterial species, in goats’ rumen [8]. Zhang et al. [9] reported that the reduction in *Ruminococcus flavefaciens* may be found when the concentration of PUFA is found at high levels. Conversely, increases in the *Ruminococus albus* population were found when both cattle [10] and goat diets [7] were supplemented with PUFA. The different findings can be attributed to the infused levels of PUFAs in the rumen [8], the specific fatty acids (type and source) [10,11,12], their bioavailability, and the animal species [8,10,13].

*Camelina sativa* is a typical example of oilseeds and a rich source of bioactive compounds such as PUFA. It has higher PUFA content compared to other oilseeds [14], the main fatty acid is α-linolenic acid (~37%) [15], and has several agronomic advantages such as lower cultivation demands [16]; thus, is considered a sustainable crop. The impact of the *Camelina sativa* and its supplementation of by-products on the rumen microbiome has been studied in the past both in vivo and in vitro, and demonstrated various significant outcomes in different ruminant species [17,18,19]. However, considering the abovementioned peculiarities (different PUFA inclusion levels in diets), it is of paramount importance to investigate the effect of different levels of *Camelina sativa* seeds (CS) supplementation in ewes’ diets on the rumen microbial population that has not been investigated on farm scale conditions. Considering the above, this is the first study aiming to evaluate the effect of the supplementation of three different levels of CS on ewes’ rumen microbiome and key microbes species related to rumen fermentation processes.

## 2. Materials and Methods

### 2.1. Experimental Diets and Experimental Design

This study was conducted respecting the approved protocols by the Ethical Committee in Research of the Agricultural University of Athens regarding animal handling, housing, and care (No. 000007/22-01-2017.). Forty-eight dairy Chios breed ewes were divided into four homogenous groups (n = 12) according to their age (2–4 years old), BW (55 ± 6.5 kg), fat corrected (6%) milk yield (FCM_6%_) (1.85 ± 0.3 kg·day^−1^), and days in milk (67 ± 8 days). Ewes were kept in a common stall but separated into different groups corresponding to each dietary treatment. At feeding time, they were trapped in individual pens to achieve individual feeding. The experimental period lasted 60 days. The ration consisted of concentrate, alfalfa hay, and wheat straw, and the average amount of each was 1.5, 1.5, and 0.2 kg/ewe/day. Furthermore, the concentrates consisted of maize grain, barley, wheat middling, sunflower meal, soybean meal, as well as mineral and vitamin premix (Table S1). Ewes were offered concentrates with the inclusion of three different levels of CS by partially substituting soybean meal and maize grain. More specifically, the concentrate of the control group (CON) had no inclusion of CS, whereas in the three following groups (CS6, CS11, and CS16), CS were included at 60, 110, and 160 g·kg^−1^ of concentrate. The average daily feed intake (g/ewe/day) and nutrient intake (g/ewe/day) of the four dietary treatments are presented in Table 1. The main fatty acid (g/ewe/day) intake is presented in Table 2. All ewes had free access to fresh water.

### 2.2. Sample Collection

Rumen samples were collected on the final day of the experiment (60th day). Rumen digesta was collected from each ewe before feeding using an esophageal tube (flexible PVG tube of 1.5 mm thickness and 10 mm I.D.) as well as a vacuum pump (MZ2CNT, Vacuubrand Gmbh & Co Kg, Wertheim, Germany). The stomach tube was inserted at a 120–150 cm depth and during the collection, the tube was moved aiming to sample from different rumen places to avoid previously reported biases [20]. The first 30 mL of rumen digesta was discarded to avoid saliva contamination which is usual when stomach tubes are used [21]. For the separation of the solid particles from the rumen fluid, four layers of cheesecloth were used. Following the sample collection, samples were snap-frozen in liquid nitrogen and then stored at −80 °C until the respective analysis.

### 2.3. Fatty Acid Determination

Individual rumen fluid samples (48 samples) were analyzed for FA composition according to the method of O’Fallon et al. [22]. For the analysis, an Agilent 6890 N gas chromatograph equipped with an HP-88 capillary column (60 m × 0.25 mm i.d. with 0.20 μm film thickness, Agilent Technologies, Inc., California, United States) and a flame ionization detector (FID) was used. The setup of the analysis, the conditions, and the extra standards used for the analysis were previously comprehensively described by Christodoulou et al. [23].

### 2.4. DNA Extraction

DNA was extracted from 96 samples (48 rumen fluid and 48 solid fractions) based on the protocol described by Mavrommatis et al. [24]. Briefly, 1 g of rumen fluid or rumen solid particles was transferred to a mortar and was ground to a powder using a pestile and nitrogen fluid. The resulting fine powder was immediately transferred to a Falcon tube into a preheated lysis buffer followed by incubation at 57 °C. RNase A was then added to each sample, followed by incubation at 37 °C. A three-fold extraction was then followed using chloroform:isoamylalcohol and before precipitation with isopropanol. The following day, after centrifugation at 7500× *g* for 15 min at 4 °C, the supernatant was discarded, followed by two ethanol washes. The DNA pellet was resuspended in ultrapure water and purified through a NucleoSpin^®^ Tissue spin column (Macherey-Nagel GmbH & Co., KG, Düren, Germany) following the manufacturer’s guidelines. The quality of the extracted DNA from each sample was tested based on the abundance of the DNA content and the impurities levels in the 260/230 and 260/280 ratios, using an ND-1000 spectrophotometer (Nanodrop, Wilmington, DE, USA), and was verified in a 0.7% agarose gel.

### 2.5. Metagenomic NGS Analysis

DNA samples, (50 ng·uL) from each rumen fluid sample, were pooled for each group (4 samples were obtained) for the metagenomic NGS bacteriome screening. The 16S rRNA gene (~1.5 kb) was amplified using a 16S Barcoding kit (SQK-RAB204, Oxford Nanopore Technologies (ONT), Oxford, UK), and following the manufacturer’s protocol. The sequencing library for 16S rRNA gene sequencing was generated from 20 ng of DNA using the same kit (SQK-RAB204 from Oxford Nanopore Technologies, Oxford, UK) following the manufacturer’s instructions and loaded into a MinION flow cell (R9.4.1, FLO-MIN106). The flow cell was placed into a MinION-Mk1B device (Oxford Nanopore Technologies) for sequencing and controlled using ONT’s MinKNOW software. Raw sequencing data (FAST5 files) were basecalled with algorithms implemented in GUPPY software (ONT) and reads were demultiplexed according to the used barcodes. Clean sequences were obtained after trimming of barcodes, adapter, and primer sequences. Processed reads (FASTQ files) were uploaded to the EPI2ME cloud-based workflow (Metrichor, Oxford, UK) for taxonomic classification of bacteria in the following major ranks: Superkingdom/phylum/class/order/family/genus/species. The analysis was carried out using NCBI ‘s ‘16S Ribosomal RNA database ‘ (bacterial and archaeal strains) with an identity threshold of 85% and a minimum quality score of 15.

### 2.6. RT-qPCR Platform for Selected Rumen Microorganisms

The primer set used for the real-time qPCR, the genomic region of PCR amplification, the primer efficiency, amplicon size, and the hybridization temperature are presented in Table S2. A description of the primer design, selection, amplification region, and validation processes is provided by Mavrommatis et al. [24].

A Step-One Plus Real-Time PCR System (Applied Biosystems, Foster City, CA, USA) was used for quantitative PCRs, with a reaction volume of 10 μL, 5 μL of SYBR^TM^ Select Master Mix (Thermo Fisher Scientific), 4 μL of primers (each 0.2 μmol), and 1 μL of DNA (20 ng·uL) as the template. By using dissociation curve analysis, primer specificity and primer dimer formation were investigated (melt curve). Based on Carberry et al. [25] equation:

(1) $$\mathrm{relative}\;\mathrm{abundance}\;=\;e\;{\left(\mathrm{target}\right)}^{\left(\mathrm{Ct}\;\mathrm{target}\;\mathrm{microorganism}-\mathrm{Ct}\;\mathrm{of}\;\mathrm{bacterial}\;16S\;\mathrm{rDNA}\right)}$$

the proportion of total bacterial 16S ribosomal DNA used to indicate the relative abundance of microbial populations. Provided that comparisons across treatments are limited and no other taxa are compared, the relative abundance expression of the results is a feasible and reliable method [26].

### 2.7. Statistical Analysis

Statistical analysis was carried out using IBM SPSS Statistics for Windows (IBM Corp. Released 2016. IBM SPSS Statistics for Windows, Version 24.0. Armonk, NY, USA). One-way ANOVA analysis was carried out to compare the effect of the dietary treatment (CON vs. CS6 vs. CS11 vs. CS16) on the relative abundance of the targeted microorganisms.

Lavene’s test was used to assess the homogeneity of the dataset and the Shapiro–Wilk test was used to test the dataset’s normality. For the data that did not violate the homogeneity and normality tests, post hoc analysis was carried out considering the Tukey multiple range tests, whereas for the data that violated these criteria, the Games–Howell test was considered. The significance threshold for these tests was set at 0.05. GraphPad Prism 8.4.2. was used for the interleaved bars and error bars represent the standard error mean (SEM).

## 3. Results

### 3.1. Rumen Fatty Acid Profile

The differences between means of the fatty acid (FA) profile in ewes’ rumen fluid of the four dietary treatments and the SEM are presented in Table 3. The concentration of C16:0 in the rumen fluid of the CS6 ewes was significantly increased (*p* = 0.007) compared with the CS11 and CS16. The concentration of cis-9 C18:1 and C18:3 n-3 were significantly increased (*p* = 0.001 and *p* = 0.003, respectively) in the CS11 and CS16 compared to the CON and CS6.

### 3.2. 16S Amplicon Sequencing

Metagenomic sequencing of DNAs isolated from pooled rumen fluid samples resulted in the detection of 19, 19, 18, and 15 phyla, 50, 57, 53, and 44 families, and 108, 139, 126, and 86 genera for CON, CS6, CS11, and CS16, respectively considering only OTUs with more than 7 reads. A minimum similarity threshold of 85% and minimum quality score of 15 were set, and approximately 100,000 reads/sample were assigned to various taxonomic levels with an average identity score of 90%. Based on the rarefaction curves of the bacterial population at the genus taxonomic level, samples reached the plateau phase. An additional increase in the number of sequences could not affect the number of genera revealed. The dominant rumen fluid phyla were Bacteroidetes, Firmicutes, and Proteobacteria. The relative abundance of Bacteroidetes was higher in the CS16 (46.6%) compared to the rest groups (CON: 39.3%; CS6: 35.0%; and CS11: 34.2%, respectively). In contrast, the relative abundance of Firmicutes was found to be higher in the CS6 (56.4%) compared with the rest (CON: 51.0%; CS11: 45.7%; and CS16: 37.6%, respectively). Regarding Proteobacteria, the highest relative abundance was found in the CS11 (18.6%) compared to the rest (CON: 7.7%; CS6: 6.5%; and CS16: 14.2%, respectively). The Firmicutes:Bacteroidetes ratio was found to be at the lowest level in the CS16 compared to the rest (Table 4). 

**Figure 1:**
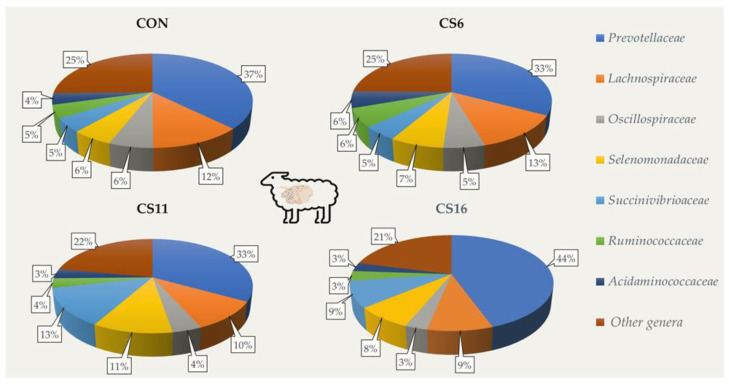
Relative abundance (%) of dominant families based on the 16S amplicon sequencing in the rumen fluid of the four pooled DNA samples representative of the four dietary treatments (CON; CS6; CS11; and CS16) with four different *Camelina sativa* seeds supplementation levels (0, 60, 110, and 160 g·kg^−1^ of concentrate, respectively) at a family level.

At a family level, *Prevotelaceae* was the dominant in the four dietary treatments (CON: 37.4%; CS6: 32.8%; CS11: 33.1%; CS16: 44.5%, respectively), followed by *Lachnospiraceae* in the CON and CS6, and by *Succinivibrioaceae* in the CS11 and CS16 (Figure 1).

**Figure 2:**
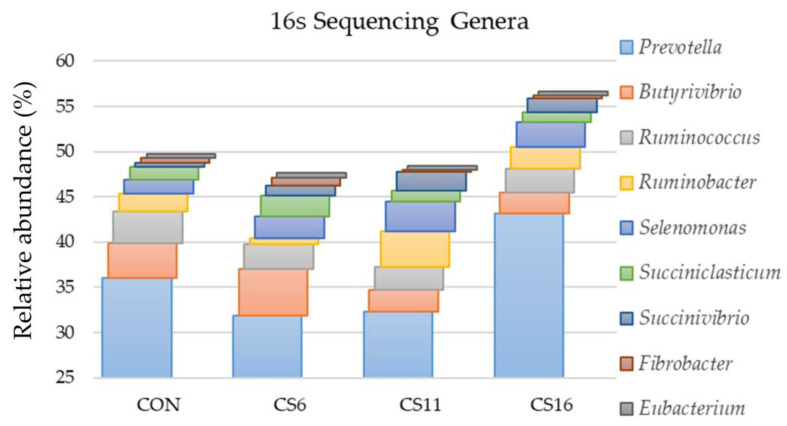
Relative abundance (%) of dominant genera based on the 16S amplicon sequencing in the rumen fluid of the four pooled DNA samples representative of the four dietary treatments (CON; CS6; CS11; and CS16) with four different *Camelina sativa* seeds supplementation levels (0, 60, 110, and 160 g·kg^−1^ of concentrate, respectively) at a genus level.

Bacteria of the genus *Prevotella* were the main representatives of the *Prevotelaceae* family in the four dietary treatments. The highest percentage was found in the CS16 (43.1%) compared with the CON (36.0%), CS6 (31.9%), and CS11 (32.3%). The predominant species of this genus was *Prevotella ruminocola* for the four dietary groups. In addition, the genus *Butyrivibrio* was the second most dominant in the CON, whereas *Ruminococcus*, *Ruminobacter*, and *Selenomonas* followed in the CS6, CS11, and CS16, respectively (Figure 2). 

### 3.3. Relative Abundance of Selected Microorganisms in the Rumen Fluid Samples Using a RT-qPCR Platform

**Figure 3:**
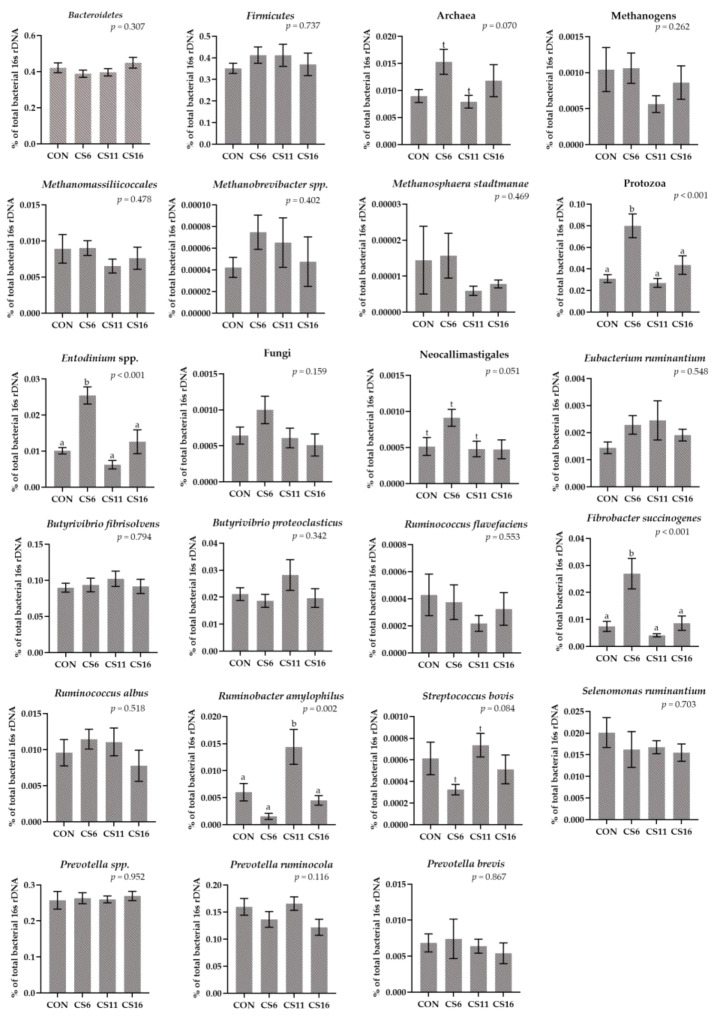
Column bar plots (±Standard error) of the average changes (*n* = 12/group) of target microorganisms as a proportion of the total rumen bacterial 16S rDNA in the rumen fluid of ewes-fed diets (CON, CS6, CS11, and CS16) with different levels of *Camelina sativa* seeds supplementation (0, 60, 110, and 160 g·kg^−1^ of concentrate, respectively). Superscript letters (a, b) in bars indicate significant differences (*p* < 0.05) between the dietary treatments, and t is referred to values between 0.05 and 0.100. Analysis was conducted using one-way ANOVA and when appropriate post hoc analysis was carried out using Tukey’s multiple-range test.

The relative abundance of Bacteroidetes and Firmicutes did not significantly differ among the four dietary groups. The Firmicutes:Bacteroidetes ratio was numerically increased in the CS6 and CS11 (Figure 3, Table S3). The relative abundance of Protozoa was significantly (*p* < 0.001) increased in the CS6 by 61.25, 66.25, and 45%, compared to the CON, CS11, and CS16, respectively (Figure 3, Table S3). A significant increase (*p* < 0.001) was also observed in the relative abundance of *Entodinium* in the CS6 (Figure 3, Table S3). Additionally, the relative abundance of Archaea tended to increase in the same dietary treatment compared to the CS11 (Figure 3, Table S3). Although a numerical decrease was observed in the relative abundance of the Methanogens in the CS11 and CS16, it was not statistically significant (Figure 3, Table S3). In addition, the relative abundance of *Ruminobacter amylophilus* was significantly increased (*p* = 0.002) in the CS11, whereas that of *Fibrobacter succinogenes* was significantly increased (*p* < 0.001) in the CS6 (Figure 3, Table S3).

### 3.4. Relative Abundance of Selected Microorganisms in the Rumen Solid Particle Using RT-qPCR Platform

**Figure 4:**
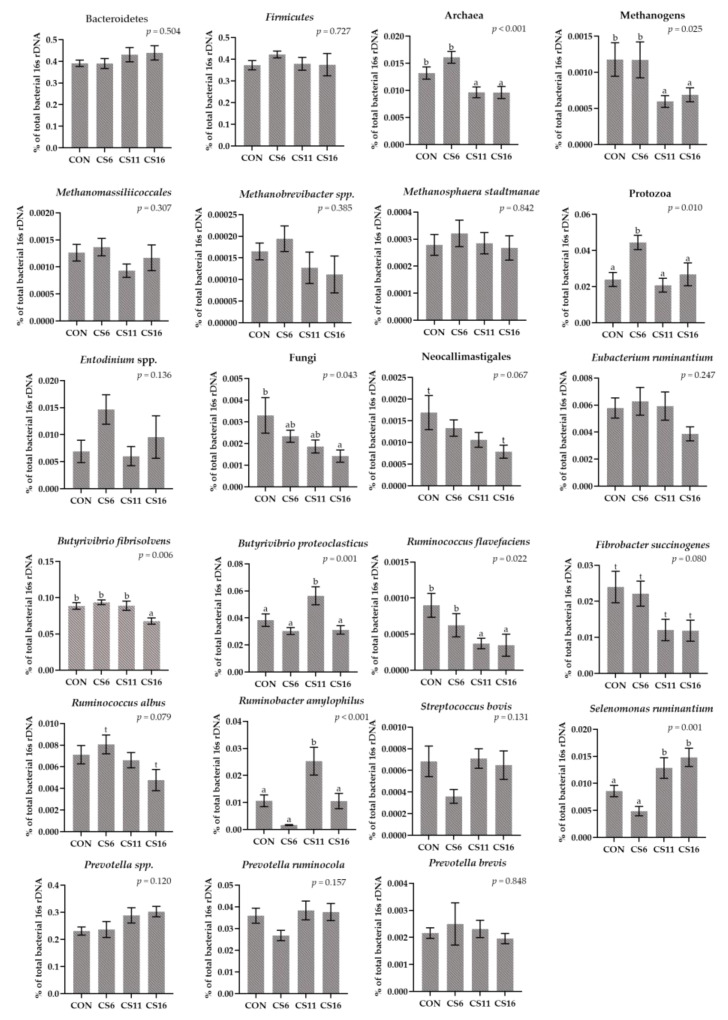
Column bar plots (±Standard error) of the average changes (*n* = 12/group) of target microorganisms as a proportion of the total rumen bacterial 16S rDNA in the rumen solid particles of ewes-fed diets (CON, CS6, CS11, and CS16) with different levels of *Camelina sativa* seeds supplementation (0, 60, 110, and 160 g·kg^−1^ of concentrate, respectively). Superscript letters (a, b) in bars indicate significant differences (*p* < 0.05) between the dietary treatments, and t is referred to values between 0.05 and 0.100. Analysis was conducted using one-way ANOVA and when appropriate post hoc analysis was carried out using Tukey multiple range test.

The relative abundance of Bacteroidetes and Firmicutes did not differ significantly among the four dietary treatments (Figure 4, Table S4). In contrast, the ratio in the relative abundance of Firmicutes:Bacteroidetes was found to be numerically reduced in the CS11 and CS16 compared with the CS6 (Figure 4, Table S4). The relative abundance of Protozoa increased significantly (*p* = 0.010) in the CS6 by 87.5, 114.3, and 66.7%, compared with the CON, CS11, and CS16, respectively (Figure 4, Table S4). A downward trend was observed in the relative abundance of *Neocallimastigales* in the group with the highest inclusion level of CS (CS16) compared to the CON (Figure 4, Table S4). Furthermore, the relative abundances of Archaea (*p* < 0.001) and total Methanogens (*p* = 0.025) were significantly decreased in the CS11 and CS16 compared with the CON and CS6 (Figure 4, Table S4). The relative abundance of *Ruminococcus flavefaciens* was significantly reduced (*p* = 0.022) in the CS11 and CS16 compared with the CON. The relative abundance of *Ruminobacter amylophilus* was significantly increased (*p* < 0.001) in the CS11, whereas the relative abundance of *Ruminococcus albus* tended towards a significant increase in the CS6 compared with the CS16 (Figure 4, Table S4). Moreover, the relative abundance of *Butyrivibrio fibrisolvens* was significantly decreased in the CS16 (*p* = 0.006), whereas *Butyrivibrio Proteoclasticus* was significantly increased in the CS11 (*p* = 0.001) (Figure 4, Table S4). Finally, the relative abundance of *Fibrobacter succinogenes* tended to significantly increase in the CS6, whereas that of *Selenomonas ruminantium* was significantly increased (*p* = 0.001) in the CS11 and CS16 compared with the CON and CS6 (Figure 4, Table S4).

## 4. Discussion

In our previous work, it was concluded that *Camelina sativa* seeds dietary supplementation did not affect ewes’ milk yield; however, it significantly reduced milk fat in the group with the highest supplemented level (160 g·kg^−1^ of concentrate, CS16) [23].

Up until now, the effect of *Camelina sativa* and its by-products supplementation on altering the rumen microbiome focused mainly on in vitro trials. However, to our knowledge, this is the first in vivo study to investigate the effect of supplementing different levels of CS on ewes’ rumen microbiome and more precisely in specific microorganisms that are crucial in the feed degradation as well as methane formation, in both fluid and solid particles of the rumen by simultaneously exploiting outcomes from both NGS and qPCR approaches. In our previous work [24], the initial screening of rumen bacteriome using an Ion Torrent 16S sequencing followed by individual RT-qPCR validation encouraged the in-depth understanding of alterations in the rumen habitat of goats fed also with PUFA-rich diets. In the present study, the advent of rapid and robust long-read sequencing by Oxford Nanopore was also used aiming to holistically assess the effect of CS supplementation in ewes providing us with a preliminary screening of rumen bacteriome. Moreover, Nanopore sequencing (Oxford Nanopore Technologies) is the most used technique for long-read sequencing, made microbial genome sequencing more accessible, and involves the facility to sequence whole genomes or specific genomic regions, whereas there have been considerable advances in accuracy [27]. After the initial bacteriome cataloging, selective rumen microorganisms’ relative abundance was assessed using a well-assessed RT-qPCR platform. 

Although there were no controversial outcomes between NGS and RT-qPCR results, some numerical discrepancies were observed; e.g., the relative abundances of *Prevotella* and *Butyrivibrio* in the rumen fluid. These fluctuations may be attributed to the different analytical workflows (pooled DNA samples on NGS vs. individual samples on RT-qPCR) and the disparate amplification region within the 16S rRNA (long-read sequencing in NGS vs. short amplicon targeting in RT-qPCR). 

### 4.1. The Effect of Camelina Sativa Seeds Supplementation in Rumen Fatty Acids Profile

A PUFA-rich diet can result in the inhibition of rumen biohydrogenation leading to severe milk fat depression (MFD). Although in the milk of the CS16 group fat was significantly reduced, milk concentration of C18:0 was increased [23]; thus, we can assume that rumen biohydrogenation was not severely impaired. However, stearic acid was numerically suppressed, whereas vaccenic acid slightly accumulated in the rumen fluid of CS16 ewes indicating a linkage between a minor inhibition of rumen biohydrogenation and MFD in CS16. The increase in the concentration of cis-9 C18:1 and C18:3 n-3 in the rumen fluid of the CS11 and CS16 ewes was due to the higher intake of these FA from the diets. Furthermore, the pattern of the C18:1 and C18:2 isomers formed during biohydrogenation in ruminants is highly dependent on the dietary fatty acid profile [28]. More specifically, the dietary inclusion of canola in dairy cows (35 g·kg^−1^ DM) increased the concentration of the C18:1 *trans* isomers compared with cows fed soy or both soy and canola [28]. 

### 4.2. The Camelina Sativa Seeds Supplementation Demonstrated Important Findings in the Modification of the Rumen Microorganisms

As expected, through the NGS technique, Bacteroidetes, Firmicutes, and Proteobacteria were the phyla that dominated ewes’ rumen bacteriome [29,30]. Considering the RT-qPCR analysis, the Firmicutes:Bacteroidetes ratio was found numerically increased in the CS6 and CS11 compared to the CON and CS16. The significant decrease in milk fat content of the CS16 ewes may be justified by the results of the Firmicutes:Bacteroidetes ratio. Similar results were also found through a DHA-rich microalgae supplementation in goats’ diets [24] and by Jami et al. [30], who demonstrated a positive correlation between the ratio of Firmicutes:Bacteroidetes and cows’ milk fat content. On the other hand, the inclusion of camelina oil in the rumen fluid of buffaloes (in vitro) increased the Firmicutes:Bacteroidetes ratio [18]. However, it is crucial to point out that the dynamic host–microbiome interplay is overlooked in the in vitro trials; thus, the results between in vivo and in vitro experiments may be controversial.

Noteworthy the highest inclusion levels of CS in ewes’ diets (CS11 and CS16), numerically decreased the relative abundance of methanogens in ewe’s rumen fluid and significantly reduced them in the solid particles, which can be considered a key topic for further investigation as it unveils important findings linked to the livestock’s environmental footprint. Our results comply with the finding of Ebeid et al. [18], who also reported a significant reduction in the buffaloes’ rumen methanogens when *Camelina* oil was ingested in vitro. In contrast, ingesting *Camelina* oil directly through the rumen cannula in dairy cattle did not significantly affect the total number of methanogens [17]. The discrepancies amongst studies may be attributed to the different *Camelina sativa* dietary supplementation types (seeds vs. oil), different dietary strategies (altering forage to concentrate ratio), administration route (diet, cannula, in vitro), as well as animal species. Another important factor generating inconsistencies between studies may lie in the methanogens’ determination method since the selected amplicon region could lead to diverse outcomes. In our study, Methanogens were targeted for amplification in the methyl coenzyme-M reductase subunit A (*MCRA*) aiming to reflect their metabolic mark through their DNA footprint. This enzyme complex is distinct and ubiquitous in methanogens; thus, it is an efficient tool for their sole detection [31].

The research on rumen methanogens has attracted great interest mainly because ruminant CH_4_ emissions contribute to greenhouse gas emissions and represent a loss of nutritional energy [32]. It has been proven that feeding ruminants with feedstuffs rich in total fat reduce CH_4_ emissions [33,34]. Moreover, MCFA and PUFA may result in a reduction in the abundance of metabolic activities of the rumen methanogens and may cause modifications in the species composition [33,35,36]. The cell membrane can be broken down by MCFA and PUFA, eliminating its selective permeability which is essential for the survival and growth of methanogens and other microorganisms. [11]. Another explanation for methanogen’s suppression may lie in unsaturated fatty acids’ toxic action against the biofilm formation in Gram-positive bacteria [37]. Therefore, if the biofilm is dispersed, rumen bacteria populations may switch back to a planktonic state, rendering them more vulnerable to abiotic influences. Although there is ample evidence about the involvement of methanogens in rumen formation, their abundance is not strictly correlated with the methane emissions per se since it has been reported that the composition of the rumen methanogens, rather than their absolute abundance, is closely related to CH_4_ production [38]. Considering not only that in vivo and in vitro outcomes may differ, but also that methanogens’ absolute abundance is not strictly correlated with the ruminants’ methane emission, future studies should combine the assessment of rumen archaeome and daily methane emissions aiming to bridge the aforementioned scientific gap. 

Notwithstanding, methanogens coexist in symbiotic interactions not only with protozoa [39] but also, were recently reported to be associated with anaerobic fungi as well [40]. Current concerns about the impact of livestock on greenhouse gas emissions led researchers to evaluate nutritional strategies for the manipulation of the rumen protozoa, to reduce CH_4_ production. It has been reported that a high content of fat may be a solid strategy for the neutralization of rumen protozoa [41]. The antiprotozoal effect of lipids depends on the composition of the FA with MCFA being more effective than PUFA in controlling protozoal numbers [42]. However, so far, no reliable and applicable method has been developed for the control of rumen protozoa, however, a series of plant extracts capable of controlling if not completely causing defaunation has been reported [43]. There is a linear relationship between protozoa concentration and CH_4_ emissions [42] and it has been estimated that up to 37% of CH_4_ production by ruminants can be attributed to methanogens associated with rumen protozoa [44,45]. However, it is important to mention that the majority of non-specific strategies for defaunation, holistically impair the rumen microbiome and consequently rumen fermentation potential. Hence, methane mitigation based on such dietary strategies is majorly a plasmatic observation that is attributed to the general suppression of rumen degradative potential. Additionally, supporting the aforementioned and the degradative importance of protozoa, a meta-analysis by Newbold et al. [43] indicated that in defaunation trials, rumen organic matter digestibility and specifically NDF and ADF digestibility were significantly decreased (−7%, −20%, and −16%, respectively) due to the loss of protozoal fibrolytic activity. In our study, the relative abundance of Protozoa was significantly increased, both in rumen fluid and solid particles of the CS6 ewes, and, consequently, this increase was also reported in the *Entodinium*. Supplementing cow diets with *Camelina* oil did not affect the total number of protozoa [17], whereas ingesting buffaloes’ rumen fluid with *Camelina* oil in different forage-to-concentrate ratios significantly decreased rumen protozoa populations in vitro [18]. Interestingly, supplementing oilseeds with high content of linoleic and a-linolenic acids in cow diets, reduced the overall population of protozoa in their rumen fluid [10]. Similarly, feeding crushed sunflower seeds and crushed canola seeds significantly decreased the rumen protozoa population in cows [46].

Furthermore, there is an interdependence between Archaea/methanogens and Fungi, as Archaea naturally attach to anaerobic fungi increasing their activity [47]. Anaerobic fungi can influence the rest of the microbial population as they produce H_2_ during the initial degradation of cell walls, which can be used as a substrate for the degradation mechanisms of other populations [48]. Investigating the effect of different feeding strategies on both the rumen fluid and solid particles is important for the detection of fungal species as while most fungi associated with celluloses and hemicelluloses are retained in the solid phase, the fluid phase may also contain smaller particles to which fungi may be attached [48]. In our study, the relative abundance of Fungi was significantly reduced in the solid but not in the fluid phase of the CS16 rumen samples. Maia et al. [49] reported that PUFA have also toxicity effects against rumen anaerobic fungi in vitro; thus, their reduction could be partly justified. Their inconsistency between rumen fluid and solid fraction could be attributed to the source of PUFA in our study (seeds). More specifically, the inclusion of PUFA-rich seeds may be related to slower PUFA release after fibrolytic species action in the seeds’ cell wall. Since the fibrolytic activity is majorly taking place in the feed particle fraction of rumen digesta, the released PUFA may severely affect fungi species also adhered to feed particles. 

The main bacterial species with the strongest cellulolytic processes are the *Fibrobacter succinogenes*, *Ruminococcus albus*, and *Ruminococcus flavefaciens* [50]. More specifically, *Fibrobacter succinogenes* and *Ruminococcus albus* are the two bacterial species that compared to the rest cellulolytic species degrade and break down cellulose faster and to a greater extent [8,51]. However, due to PUFA toxicity, the relative abundance of such bacterial species may decrease [49,52,53]. More specifically, the administration of oilseeds, due to their PUFA content, has been shown to reduce bacterial species with cellulolytic activity [8,19]. In this study, the relative abundance of *Ruminococcus flavefaciens* was significantly decreased in the rumen solid particles of the ewes of the CS11 and CS16, which agrees with the work of Dai et al. [19], who reported a reduced abundance of *Ruminococcus* spp., *Fibrobacter* spp., and *Butyriviprio* spp. in vitro due to CS supplementation. In compliance with our findings, supplementing the whole linseed in goat diets at 10 and 20% significantly reduced the populations of *Fibrobacter succinogenes* and *Ruminococcus flavefaciens* but not *Ruminococcus albus* [8]. On the contrary, a significant increase was observed in the relative abundance of *Fibrobacter succinogenes* in the rumen fluid of the group with the lowest supplementation level of CS (CS6). In addition, Bayat et al. [17] did not report any significant alteration when dairy cow diets were supplemented with *Camelina* oil. In contrast, although in dairy cows’ rumen fluid, the administration of oilseeds especially rich in linoleic acid decreased the population of *Fibrobacter succinogenes*, it increased those of *Ruminococcus albus* and *Ruminococcus flavefaciens* [10]. Authors suggested that the measured cellulolytic bacteria were at the expense of other bacterial populations [10]. Although the three dominant bacterial species with prominent fibrolytic activity showed a decreasing trend in the solid fraction of ewes’ rumen with the inclusion of CS, no significant alterations were observed for animal performance [23] or health status [23,54]. However, further studies should be carried out assessing the impact of CS in rumen enzymatic degradative potential and apparent total tract digestibility indices aiming to clarify if the aforementioned microbes’ fluctuations could cause functional consequences.

Moreover, there are non-cellulolytic bacteria such as *Treponema bryantii* [55] and *Prevotella ruminicola* [56] that are important for cellulose degradation, since they can activate cellulolytic bacteria through the “cross-feeding” interaction [57] and cooperate with cellulolytic bacteria to improve cellulose digestion. Interspecies H_2_ transport and metabolite removal and/or exchange are factors thought to contribute to such synergy [57]. One of the most important non-cellulolytic bacterial species that are involved in cellulolytic processes is *Selenomonas ruminantium*. It improves cellulose digestion when co-cultured with *Ruminococcus flavefaciens* by converting succinic acid, a metabolite of *Ruminococcus flavefaciens*, to propionate [58]. A similar relationship was hypothesized for the combination of *Selenomonas ruminantium* and *Fibrobacter succinogenes* [59], and it was found that their combination resulted in a synergistic increase in propionate production [60]. Evaluating this synergy is necessary to maximize cellulose digestion in the rumen as *Fibrobacter succinogenes*, as previously mentioned, is considered the most important cellulolytic species in this direction [51]. Notably, supplementing CS at the two highest inclusion levels (CS11 and CS16) significantly increased the relative abundance of *Selenomonas ruminantium*, which agrees with an in vitro study of a dual-flow continuous culture system that evaluated two different levels of CS supplementation [19]. These results lead to the conclusion that supplementing CS in ewes; diets may balance the population of bacterial species involved in cellulose degradation and digestion. Therefore, the inverse modifications observed in the relative abundance of *Ruminococcus flavefaciens*, *Selenomonas ruminantium,* and *Fibrobacter succinogenes* in both rumen fluid and solid particles of the four dietary treatments may indicate balance in bacterial populations involved in cellulolytic processes.

Furthermore, there are bacterial species responsible for the degradation of non-structural polysaccharides or starch (amylolytic) and proteins (proteolytic). *Ruminobacter amylophilus*, *Butyrivibrio fibrisolvens*, and *Streptococcus bovis* are involved in both degradation processes, whereas *Prevotella* sp. and *Megasphaera elsdenii* demonstrate strong proteolytic ability [61]. Interestingly, the relative abundance of species of the genus *Prevotella* was not significantly affected in our study. As previously mentioned, the relative abundance of *Ruminobacter amylophilus* was significantly increased in the rumen fluid of the CS11. The NGS analysis confirmed this finding, showing a greater abundance in the same group. *Butyrivibrio fibrisolvens* is also considered responsible for the biohydrogenation and is involved in its initial stages in which PUFA are biohydrogenated to trans C18:1. On the other hand, *Butyrivibrio proteoclasticus* biohydrogenates trans C18:1 to C_18:0_. The supplementation of CS did not significantly affect the relative abundance of the aforementioned bacterial species in the rumen fluid; however, in the solid particles, it caused a decrease in the relative abundance of the *Butyrivibrio fibrisolvens* in the CS16, which may be attributed to the highest inclusion level PUFA toxicity [24], and an increase in the relative abundance of the *Butyrivibrio proteoclasticus* in the CS11. However, these alterations did not affect at this pattern the rumen’s FA profile.

## 5. Conclusions

Although the initial screening was carried out using NGS technology followed by specific microbes targeting unveiled important changes in the rumen microbiota under CS supplementation levels, in the era of omics techniques, more information is needed in order to deeply understand the impact of CS and dietary PUFA on rumen biochemistry by screening numerous taxa and their functional reflection. The dietary supplementation with 160 g·kg^−1^ CS in ewes’ concentrates induced favourable changes in methanogen populations, whereas changes in bacterial species related to degradation processes in the rumen might not affect animal performance but are accombinied by a reduction in milk fat content. 

## Figures and Tables

**Table 1:** Average daily feed intake (g/ewe/day) and nutrients intake (g/ewe/day) of the four dietary treatments (CON, CS6, CS11, CS16) with different levels of *Camelina sativa* seeds (0, 60, 110, and 160 g·kg^−1^ of concentrate).

Daily Feed Intake (g/Ewe/Day as Fed)	Dietary Treatments (D)			
	CON	CS6	CS11	CS16
Wheat Straw	200	200	200	200
Alfalfa Hay	1500	1500	1500	1500
Concentrate	1500	1500	1500	1500
Nutrients intake (g/ewe/day)	Dietary treatments (D)			
	CON	CS6	CS11	CS16
Dry Matter	2880	2874	2882	2888
Crude Protein	601	600	602	620
Ether Extract	40	80	107	133
Neutral Detergent Fiber	1284	1285	1287	1301
Acid Detergent Fiber	665	711	712	719

CON: dietary treatment with 0 g *Camelina sativa* seeds·kg^−1^ of concentrate. CS6: dietary treatment with 60 g *Camelina sativa* seeds·kg^−1^ of concentrate. CS11: dietary treatment with 110 g *Camelina sativa* seeds·kg^−1^ of concentrate. CS16: dietary treatment with 160 g *Camelina sativa* seeds·kg^−1^ of concentrate.

**Table 2:** Main fatty acid intake (g/ewe/day) of the four dietary treatments (CON, CS6, CS11, CS16) with different levels of *Camelina sativa* seeds (0, 60, 110, and 160 g·kg^−1^ of concentrate).

	Dietary Treatments (D)			
Main Fatty Acids Intake (g/Ewe/Day) of the Total Diet	CON	CS6	CS11	CS16
C14:0	0.18	0.24	0.27	0.25
C16:0	7.50	9.57	10.74	11.78
C16:1 n-7	0.20	0.23	0.25	0.27
C18:0	1.34	2.14	2.62	3.08
cis-9 C18:1	7.47	13.57	18.35	22.41
cis C18:2 n-6	19.94	29.08	31.74	37.67
C20:0	0.11	0.33	0.51	0.67
C18:3 n-3	2.02	14.40	23.01	30.88
C20:1 n-9	0.21	6.71	11.41	15.63
C20:2 n-6	0.16	0.85	1.38	1.80
C20:3 n-3	0.14	0.24	0.34	0.41
C22:0	0.07	0.13	0.18	0.16
C24:0	0.22	0.26	0.31	0.37

CON: dietary treatment with 0 g *Camelina sativa* seeds·kg^−1^ of concentrate. CS6: dietary treatment with 60 g *Camelina sativa* seeds·kg^−1^ of concentrate. CS11: dietary treatment with 110 g *Camelina sativa* seeds·kg^−1^ of concentrate. CS16: dietary treatment with 160 g *Camelina sativa* seeds·kg^−1^ of concentrate.

**Table 3:** The mean individual fatty acids (FA) (% of total FA) in ewes’ rumen fluid of the four dietary treatments (CON, CS6, CS11, and CS16) with different levels of *Camelina sativa* seeds (0, 60, 110, and 160 g·kg^−1^ of concentrate).

	Dietary Treatment					
Fatty Acid	CON	CS6	CS11	CS16	SEM ^a^	*p*
C14:0	1.22	0.91	0.72	0.57	0.11	0.235
C14:1	1.51	1.26	0.88	1.00	0.11	0.180
C15:0	0.86	0.87	1.02	0.94	0.08	0.887
C16:0	24.33 ^ab^	29.32 ^b^	21.09 ^a^	21.84 ^a^	0.10	0.007
C16:1 n-7	0.26	0.04	0.09	0.18	0.04	0.310
C17:0	0.22	0.00	0.04	0.10	0.03	0.138
C18:0	46.37	42.78	45.07	42.18	0.87	0.308
trans C18:1	0.82	1.16	1.68	1.39	0.24	0.644
trans-11 C18:1	5.93	5.32	8.01	7.78	0.50	0.137
cis-9 C18:1	8.03 ^a^	9.02 ^a^	11.84 ^b^	12.40 ^b^	0.49	0.001
trans C18:2 n-6	0.00	0.00	0.06	0.07	0.02	0.622
cis C18:2 n-6	5.91	4.71	4.58	4.73	0.26	0.265
C20:0	0.39	0.50	0.23	0.80	0.14	0.275
C18:3 n-3	0.48 ^a^	0.59 ^a^	1.53 ^b^	1.30 ^b^	0.13	0.003
cis-9, trans-11 C18:2	3.54	3.32	2.55	3.71	0.26	0.395
C20:2 n-6	0.11	0.10	0.05	0.22	0.04	0.413
C20:4 n-6	0.38	0.16	0.56	0.81	0.14	0.445

Means with different superscript letters (a, b) between dietary groups differ significantly at *p* < 0.05. ^a^ SEM: Standard error of the means. CON: dietary treatment with 0 g *Camelina sativa* seeds·kg^−1^ of concentrate (*n* = 12). CS6: dietary treatment with 60 g *Camelina sativa* seeds·kg^−1^ of concentrate (*n* = 12). CS11: dietary treatment with 110 g *Camelina sativa* seeds·kg^−1^ of concentrate (*n* = 12). CS16: dietary treatment with 160 g *Camelina sativa* seeds·kg^−1^ of concentrate (*n* = 12).

**Table 4:** Relative abundance (%) of the metagenomic sequencing of the genomic DNA of the pooled rumen fluid samples from the four dietary treatments (CON, CS6, CS11, and CS16) with different levels of *Camelina sativa* seeds (0, 60, 110, and 160 g·kg^−1^ of concentrate).

Dietary Treatments	Phyla (Relative Abundance,%)			
	*Bacteroidetes*	*Firmicutes*	*Proteobacteria*	*Firmicutes: Bacteroidetes*
CON	39.3	51.0	7.7	1.30
CS6	35.0	56.4	6.5	1.61
CS11	34.2	45.7	18.6	1.34
CS16	46.7	37.6	14.2	0.81

CON: dietary treatment with 0 g *Camelina sativa* seeds·kg^−1^ of concentrate (*n* = 1; 12 DNA samples pooled). CS6: dietary treatment with 60 g *Camelina sativa* seeds·kg^−1^ of concentrate (*n* = 1; 12 DNA samples pooled). CS11: dietary treatment with 110 g *Camelina sativa* seeds·kg^−1^ of concentrate (*n* = 1; 12 DNA samples pooled). CS16: dietary treatment with 160 g *Camelina sativa* s seeds·kg^−1^ of concentrate (*n* = 1; 12 DNA samples pooled).

## Data Availability

All data are available within the manuscript and Supplementary Materials.

## Supplementary Materials

The following supporting information can be downloaded at: https://www.mdpi.com/article/10.3390/ani13030377/s1, Table S1: Concentrate ingredients of the four dietary treatments (CON, CS6, CS11, CS16) with different levels of *Camelina sativa* seeds (0, 60, 110, and 160 g·kg^−1^ of concentrate); Table S2: Sequences of primers used for RT-PCRs, genomic regions of PCR amplification, primer efficiency, amplicon size, and hybridization temperature; Table S3: Relative abundance of the microorganisms in ewes’ rumen fluid of the four dietary treatments (CON, CS6, CS11, CS16) with different levels of *Camelina sativa* seeds (0, 60, 110, and 160 g·kg^−1^ of concentrate); Table S4: Relative abundance of the microorganisms in ewes’ rumen solid of the four dietary treatments (CON, CS6, CS11, CS16) with different levels of *Camelina sativa* seeds (0, 60, 110, and 160 g·kg^−1^ of concentrate).
