# Supplementary material for: Inclusion of Camelina sativa Seeds in Ewes’ Diet Modifies Rumen Microbiota
Source: Animals (Basel). 2023 Jan 22;13(3):377. doi: 10.3390/ani13030377 (PMC9913825; doi:10.3390/ani13030377)
Supplement: Supplementary file 1 [file animals-13-00377-s001.zip › animals-2064530-supplementary.pdf]

animals

MDPI

Table S1. Concentrate ingredients of the four dietary treatments (CON, CS6, CS11, CS16) with different levels of *Camelina sativa* seeds (0, 60, 110, and 160 g·kg^{-1} of concentrate).

Ingredients (g/kg)	Concentrates			
	CON	CS6	CS11	CS16
Camelina sativa seeds	0	60	110	160
Maize grain	344	299	269	239
Barley	200	200	200	200
Wheat middlings	100	100	100	100
Sunflower meal	160	180	180	180
Soybean meal	155	120	100	80
Premix mineral and vitamins	41	41	41	41

CON: dietary treatment with 0 g *Camelina sativa* seeds·kg^{-1} of concentrate; CS6: dietary treatment with 60 g *Camelina sativa* seeds·kg^{-1} of concentrate; CS11: dietary treatment with 110 g *Camelina sativa* seeds·kg^{-1} of concentrate; CS16: dietary treatment with 160 g *Camelina sativa* seeds·kg^{-1} of concentrate.

Table S2. Sequences of primers used for RT-PCRs, genomic regions of PCR amplification, primer efficiency, amplicon size, and hybridization temperature.

Target Species	Genomic Region of PCR Amplification	Primer Sequencing	Primer Efficiency %	Slope	Amplicon bp	Tm °C
Total bacteria	16s rRNA	F: 5′-CGGCAACGAGCGCAACCC-3′<br>R: 5′-CCATTGTAGCACGTGTGTAGCC-3′	98	−3.378	130	60
Bacteroidetes	16s rRNA	F: 5′-GGARCATGTGGTTTAATTCGATGAT-3′<br>R: 5′-AGCTGACGACAACCATGCAG-3′	98	−3.36	126	62
Firmicutes	16s rRNA	F: 5′-GGAGYATGTGGTTTAATTCGAAGCA-3′<br>R: 5′-AGCTGACGACAACCATGCAC-3′	97	−3.39	126	62
Archaea	16s rRNA	F: 5′-GAGGAAGGAGTGGACGACGGTA-3′<br>R: 5′-ACGGGCGGTGTGTGCAAG-3′	96	−3.43	233	60
Protozoa	18s rRNA	F: 5′-GCTTTCGWTGGTAGTGTATT-3′<br>R: 5′-CTTGCCCTCYAATCGTWCT-3′	97	−3.39	223	55
Entodinium	18s rRNA	F: 5′-GAGCTAATACATGCTAAGGC-3′<br>R: 5′-CCCTCACTACAATCGAGATTTAAGG-3′	97	−3.39	317	59
Total fungi	18s rRNA ITS1	F: 5′-GAGGAAGTAAAAGTCGTAACAAGGTTTC-3′<br>R: 5′-CAAATTCACAAAGGGTAGGATGATT-3′	98	−3.38	120	58
Neocallimastigales	18s rRNA ITS1	F: 5′-TTGACAATGGATCTCTTGGTTCTC-3′<br>R: 5′-GTGCAATATGCGTTCGAAGATT-3′	96	−3.43	110	63
Methanogen	mcrA	F: 5′-TTCGGTGGATCDCARAGRGC-3′<br>R: 5′-GBARGTCGWAWCCGTAGAATCC-3′	95	−3.44	140	58
Methanomassiliicoccales	16s rRNA	F: 5′-TTCTGGGGTAGGGGTAAAATC-3′<br>R: 5′-GTCTGCAGCGTTTACACCCT-3′	97	−3.40	149	62
Methanobrevibacter spp.	16s rRNA	F: 5′-TGGGAATTGCTGGWGATACTRTT-3′<br>R: 5′-GGAGCRGCTCAAAGCCA-3′	95	−3.46	231	60
Methanosphaera stadtmanae	16s rRNA	F: 5′-CTTAACTATAAGAATTGCTGGAG-3′<br>R:5′-TTCGTTACTCACCGTCAAGATC-3′	97	−3.39	150	58
Butyrivibrio fibrisolvens	16s rRNA	F: 5′-TAACATGAGAGTTTGATCCTGGCTC-3′<br>R: 5′-CGTTACTCACCCGTCCGC-3′	97	−3.39	136	58
Butyrivibrio proteoclasticus	16s rRNA	F: 5′-TCCGGTGGTATGAGATGGGC-3′<br>R: 5′-GTCGCTGCATCAGAGTTTCCT-3′	98	−3.38	185	60
Eubacterium ruminantium	16s rRNA	F: 5′-CTCCCGAGACTGAGGAAGCTTG-3′<br>R: 5′-GTCCATCTCACACCACCGGA-3′	98	−3.36	184	62

Ruminococcus flavefaciens	16s rRNA	F: 5′-CGAACGGAGATAATTTGAGTTTACTTAGG-3′ R: 5′-CGGTCTCTGTATGTTATGAGGTATTACC-3′	95	−3.42	132	60
Fibrobacter succinogenes	16s rRNA	F: 5′-GCGGGATTGAATGTACCTTGAGA-3′ R: 5′-TCCGCCTGCCCCTGAACTATC-3′	98	−3.39	204	60
Ruminococcus albus	16s rRNA	F: 5′-CCCTAAAAGCAGTCTTAGTTCG-3′ R: 5′-CCTCCTTGCGGTTAGAACA-3′	98	−3.38	175	62
Ruminobacter amylophilus	16s rRNA	F: 5′-ATGCAAGTCGAACGGTAACAGCAGG-3′ R: 5′-GCACCCGTTTCCAGGTGTTGTCC-3′	96	−3.42	115	65
Streptococcus bovis	16s rRNA	F: 5′-TTCCTAGAGATAGGAAGTTTCTTCGG-3′ R: 5′-ATGATGGCAACTAACAATAGGGGT-3′	96	−3.43	127	57
Selenomonas ruminantium	16s rRNA	F: 5′-CAATAAGCATTCCGCCTGGG-3′ R: 5′-TTCACTCAATGTCAAGCCCTGG-3′	99	−3.35	138	57
Prevotella sp.	16s rRNA	F: 5′-GGTTCTGAGAGGAAGGTCCCC-3′ R: 5′-TCCTGCACGCTACTTGGCTG-3′	96	−3.42	121	60
Prevotella brevis	16s rRNA	F: 5′-GGTTTCCTTGAGTGTATTCGACGTC-3′ R:5′-CTTTCGCTTGGCCGCTG-3′	98	−3.38	219	64
Prevotella ruminicola	16s rRNA	F: 5′-GAAAGTCGGATTAATGCTCTATGTTG-3′ R:5′-CATCCTATAGCGGTAAACCTTTGG-3′	97	−3.39	74	63

Table S3. Relative abundance of the microorganisms in ewes′ rumen fluid of the four dietary treatments (CON, CS6, CS11, CS16) with different levels of *Camelina sativa* seeds (0, 60, 110, and 160 g·kg^{-1} of concentrate).

	Dietary treatments					
	**CON**	**CS6**	**CS11**	**CS16**	**SEM**[a]	***p***
Bacteroidetes	0.421	0.388	0.396	0.449	0.012	0.307
Firmicutes	0.351	0.413	0.412	0.370	0.022	0.737
Firmicutes:Bacteroidetes	0.844	1.055	1.047	0.806	0.048	0.137
Prevotella sp.	0.257	0.263	0.260	0.269	0.007	0.952
Prevotella ruminocola	0.160	0.136	0.166	0.122	0.007	0.116
Prevotella brevis	0.007	0.007	0.006	0.005	0.002	0.867
Protozoa	0.031^{b}	0.080^{a}	0.027^{b}	0.044^{b}	0.005	<0.001
Total Fungi	0.0006	0.0010	0.0006	0.0005	0.00008	0.159
Neocallimastigales	0.0005^{t}	0.0009^{t}	0.0005^{t}	0.0006	0.00007	0.051
Entodinium	0.010^{b}	0.025^{a}	0.006^{b}	0.013^{b}	0.002	<0.001
Archaea	0.009	0.015^{t}	0.008^{t}	0.012	0.001	0.070
Total Methanogen	0.0010	0.0010	0.0006	0.0008	0.0001	0.262
Methanomassiliicoccales	0.009	0.009	0.007	0.008	0.0007	0.478
Methanobrevibacter	<0.0001	0.0001	0.0001	<0.0001	0.00001	0.402
Methanosphaera stadtmanae	<0.0001	<0.0001	<0.0001	<0.0001	0.00000	0.469
Ruminococcus flavefaciens	0.0004	0.0004	0.0002	0.0003	0.00005	0.553
Ruminobacter amylophilus	0.006^{b}	0.002^{b}	0.014^{a}	0.004^{b}	0.0014	0.002
Ruminococcus albus	0.010	0.011	0.011	0.008	0.0009	0.518
Butyrivibrio fibrisolvens	0.090	0.094	0.102	0.092	0.005	0.794
Butyrivibrio proteoclasticus	0.021	0.019	0.028	0.020	0.002	0.342
Fibrobacter succinogenes	0.007^{b}	0.027^{a}	0.004^{b}	0.009^{b}	0.002	<0.001
Selenomonas ruminantium	0.020	0.016	0.017	0.015	0.001	0.703
Streptococcus bovis	0.0006	0.0003^{t}	0.0007^{t}	0.0005	0.00006	0.084
Eubacterium ruminantium	0.001	0.002	0.002	0.002	0.0003	0.548

CON: dietary treatment with 0 g *Camelina sativa* seeds·kg^{-1} of concentrate; CS6: dietary treatment with 60 g *Camelina sativa* seeds·kg^{-1} of concentrate; CS11: dietary treatment with 110 g *Camelina sativa* seeds·kg^{-1} of concentrate; CS16: dietary treatment with 160 g *Camelina sativa* seeds·kg^{-1} of

concentrate; Different superscript letters (a. b) between dietary treatments differ significantly ($p < 0.05$) and t is referred to values between 0.05 and 0.100 ($0.05 < t < 0.10$); [a] SEM = standard error mean; p = p value.

Table S4. Relative abundance of the microorganisms in ewes' rumen solid of the four dietary treatments (CON, CS6, CS11, CS16) with different levels of *Camelina sativa* seeds (0, 60, 110, and 160 g·kg^{-1} of concentrate).

	Dietary treatments					
	**CON**	**CS6**	**CS11**	**CS16**	**SEM**[a]	***p***
Bacteroidetes	0.391	0.390	0.431	0.439	0.014	0.504
Firmicutes	0.373	0.422	0.379	0.375	0.016	0.727
Firmicutes:Bacteroidetes	0.962	1.112	0.892	0.866	0.040	0.143
Prevotella spp.	0.231	0.236	0.289	0.303	0.013	0.120
Prevotella ruminocola	0.036	0.027	0.038	0.038	0.002	0.157
Prevotella brevis	0.022	0.025	0.023	0.020	0.0002	0.848
Protozoa	0.024^{b}	0.045^{a}	0.021^{b}	0.027^{b}	0.003	0.010
Total Fungi	0.0030^{a}	0.0023^{ab}	0.0019^{ab}	0.0014^{b}	0.0002	0.043
Neocallimastigales	0.017^{t}	0.013	0.011	0.010^{t}	0.0001	0.067
Entodinium	0.069	0.147	0.060	0.096	0.001	0.136
Archaea	0.013^{a}	0.016^{a}	0.010^{b}	0.010^{b}	0.0007	<0.001
Total Methanogen	0.0012^{a}	0.0012^{a}	0.0006^{b}	0.0007^{b}	0.0001	0.025
Methanomassiliicoccales	0.0013	0.0014	0.0009	0.0011	0.0001	0.307
Methanobrevibacter	0.00016	0.00019	0.00013	0.00011	0.00002	0.385
Methanosphaera stadtmanae	0.0003	0.0003	0.0003	0.0003	0.00002	0.842
Ruminococcus flavefaciens	0.0009^{a}	0.0006^{ab}	0.0003^{b}	0.0003^{b}	0.00007	0.022
Ruminobacter amylophilus	0.011^{b}	0.002^{b}	0.025^{a}	0.011^{b}	0.002	<0.001
Ruminococcus albus	0.071	0.081^{t}	0.066	0.048^{t}	0.0005	0.079
Butyrivibrio fibrisolvens	0.089^{a}	0.094^{a}	0.089^{a}	0.068^{b}	0.003	0.006
Butyrivibrio Proteoclasticus	0.038^{b}	0.031^{b}	0.056^{a}	0.031^{b}	0.003	0.001
Fibrobacter succinogenes	0.024^{t}	0.022^{t}	0.012^{t}	0.012^{t}	0.002	0.080
Selenomonas ruminantium	0.009^{b}	0.005^{b}	0.013^{a}	0.015^{a}	0.001	0.001
Streptococcus bovis	0.0007	0.0004	0.0007	0.0006	0.00006	0.131
Eubacterium ruminantium	0.006	0.006	0.006	0.004	0.0004	0.247

CON: dietary treatment with 0 g *Camelina sativa* seeds·kg^{-1} of concentrate; CS6: dietary treatment with 60 g *Camelina sativa* seeds·kg^{-1} of concentrate; CS11: dietary treatment with 110 g *Camelina sativa* seeds·kg^{-1} of concentrate; CS16: dietary treatment with 160 g *Camelina sativa* seeds·kg^{-1} of concentrate; Different superscript letters (a. b) between dietary treatments differ significantly ($p < 0.05$) and t is referred to values between 0.05 and 0.100 ($0.05 < t < 0.100$); [a] SEM = standard error mean; p = p value.
